# Bcl-2 Associated Athanogene 2 (BAG2) is Associated With Progression and Prognosis of Hepatocellular Carcinoma: A Bioinformatics-Based Analysis

**DOI:** 10.3389/pore.2021.594649

**Published:** 2021-04-01

**Authors:** Xi Zhang, Junjun Zhang, Yang Liu, Jie Li, Juan Tan, Zewen Song

**Affiliations:** ^1^ Department of Oncology, The Third Xiangya Hospital of Central South University, Changsha, China; ^2^ Department of Pathology, The Third Xiangya Hospital of Central South University, Changsha, China; ^3^ Department of Information Science and Engineering, Hunan University of Chinese Medicine, Changsha, China

**Keywords:** BAG2, hepatocellar carcinoma, ribosome biogenesis, invasion, apoptosis

## Abstract

**Background:** Bcl-2 associated athanogene2 (BAG2) is reported to act as an oncogene or a tumor-suppressor in tumors in a context-dependent way; however, its function in hepatocellular carcinoma (HCC) remains unclear.

**Methods:** Immunohistochemistry (IHC) staining, cell counting kit-8 (CCK-8) assay, apoptotic assay, cell invasion assay and a set of bioinformatics tools were integrated to analyze the role of BAG2 in hepatocellular carcinoma.

**Results:** BAG2 was significantly up-regulated in HCC. Prognostic analysis indicated that HCC patients with high expression of BAG2 had significantly shorter overall survival, progression free survival and disease specific survival. Besides, silencing BAG2 in HCC cells impaired cell proliferation, facilitated apoptosis and repressed invasion of the cells. Bioinformatics analysis showed that BAG2 might regulate ribosome biogenesis in HCC.

**Conclusion:** This study revealed that the up-regulated BAG2 in HCC was associated with a worse prognosis and might favor the progression of the disease.

## Introduction

Hyperactive ribosome biogenesis is a hallmark of many types of cancer including hepatocellular carcinoma (HCC) [1, 2]. Undoubtedly, increased ribosome biogenesis and protein synthesis is essential to support tumor proliferation and growth, and some recent studies indicate that aberrant regulation of ribosomes could drive tumorigenesis [1]. In addition, ribosome biogenesis is further found to be required for the epithelial-mesenchymal transition (EMT) of cancer cells during tumor invasion [3]. CX-5461, the first-in-class selective ribosome DNA (rDNA) transcription inhibitor, demonstrates anti-tumor activity against advanced hematologic cancers, with the best response being partial response (PR) sustained for more than 12 months [4]. Thus, targeting components or regulators of ribosome biogenesis represents a new therapeutic strategy in cancer treatment.

The Bcl-2 associated athanogene (BAG) family, first known as a group of proteins preventing cell death through their interaction with Bcl-2, participates in the regulation of physiological activities ranging from apoptosis to tumorigenesis [5, 6]. To date, six members of the family have been identified in humans and are named from BAG1 to BAG6. The function of these proteins have been well studied in neurodegenerative disorders like Alzheimer’s and Parkinson’s disease, but recently the family has become a focus in cancer-oriented research [5]. BAG2 shares a C-terminal conserved region (the BAG domain) with other members of the family, allowing it to bind the HSP70 family of molecular chaperones [5]. As a member of the BAG family, BAG2 is also found to play an essential role in several types of cancer [7–10]. Kyung-Min Yang et al. found that BAG2 was significantly over-expressed in triple-negative breast cancer (TNBC) and favored tumor progression by regulating pro-cathepsin B/annexin II complex formation and facilitating the secretion of pro-cathepsin B, which induces metastasis [9]. Besides, elevated level of BAG2 in tumors might cause accumulation of mutant p53 proteins that drive tumor growth, by binding to the proteins and inhibiting MDM2-mediated ubiquitination and degradation [8]. An oncogenic function of BAG2 is also reported in gastric cancer and oral cancer [7, 10]. However, BAG2 might play a pro-apoptotic role in thyroid carcinoma, because silencing the expression of the gene partially inhibits cell death in these cells exposed to the proteasome inhibitor MG132 [11]. This inconsistency indicates that BAG2 might function as an oncogene or a tumor-suppressor in tumors in a context-dependent way.

Since no study, to our knowledge, explores the function of BAG2 in HCC, it is unclear whether the gene plays a role in the disease. In this study, we found that BAG2 was significantly up-regulated in HCC, and patients with high expression of BAG2 showed a significantly shorter overall survival (OS), progression free survival (PFS) and disease specific survival (DSS). *In vitro* experiments showed that silencing BAG2 in HCC cells impaired cell proliferation, facilitated apoptosis and repressed invasion of the cells. By using a set of bioinformatics tools, we further found that BAG2 might participate in the ribosome biogenesis of HCC.

## Materials and Methods

### Cell Culture and Human Tissue

HepG2 cells were obtained from the Chinese Academy of Sciences Cell Bank (Shanghai, China), cultured in RPMI-1640 medium (Invitrogen, United States) supplemented with 10% fetal bovine serum (FBS, ExCell, China), and were incubated under humidified atmospheric conditions with 5% CO_2_ at 37°C [12]. HCC patients’ tumor (*n* = 10) and tumor-adjacent normal tissues (*n* = 10) were collected in the Third Xiangya Hospital and used for immunohistochemistry (IHC) staining of BAG2. The clinicopathological characteristics of these patients were summarized in Supplementary Table S1. Use of human tissue was approved by the institutional review board (IRB) of the Third Xiangya Hospital, Central South University (No: 2020-S374). Written informed consent form (ICF) had been obtained from patients whose tissues were used in this study.

### RNA Extraction and RT-PCR

Total RNA was extracted by using Trizol (Pufei Biotech Co., Ltd., Shanghai, China), and then quantified with the Nanodrop 1,000 (Thermo Fisher Scientific, United States), followed by cDNA synthesis (Promega, United States). 6 ul SYBR Premix Ex Taq (TAKARA, Japan), 0.3 ul primers, 0.6 ul cDNA, and 5.1 ul RNase-free H_2_O were added into 96-well plates for Real-time PCR (RT-PCR) on the LightCycler 480 II (Roche, Switzerland). The running parameters were set as: 95°C for 30 s, 40 cycles of PCR (95°C for 5 s and 60°C for 30 s), and dissociation (95°C for 15 s, 60°C for 30 s and 95°C for 15 s) [13]. The primers used in this work were obtained from Ruan Tuo Biotechnologies (Shanghai, China) and their sequences were included in Supplementary Table S2. Results were first normalized against the housekeeping gene GAPDH, which is stable across all samples, and then against the experimental controls.

### Small Interfering RNA Silencing of BAG2

Three small interfering RNAs targeting BAG2 (siBAG2), named from si-BAG2-1 to si-BAG2-3, and a negative control (NC) siRNA (si-NC) were designed by and purchased from Ruan Tuo Biotechnologies (Shanghai, China). The sequences were si-BAG2-1 (sense : 5′-GUACUAGGAUCUAGCAUAUUUTT-3′, Anti-sense : 5′-AAAUAUGCUAGAUCCUAGUACTT-3′), si-BAG2-2 (sense : 5′-GAUC-AGAAGUUUCAAUCCAUATT-3′, Anti-sense : 5′- UAU​GGA​UUG​AAA​CUU​CUG​AUC​TT-3′), and si-BAG2-3 (sense : 5′- AGC​AUG​CCA​CAA​GGA​UUA​UUG​TT-3′, Anti-sense : 5′- CAA​UAA​UCC​UUG​UGG​CAU​GCU​TT-3′), si-NC (sense : 5′- UUCUCCGAACGUGUCACGUdTdT-3′, Anti-sense : 5′- ACGUGACACGUUCGGAGAAdTdT-3′). HepG2 cells were seeded into 12-well plates, and transfection of siRNA was conducted by using Lipofectamine™ 2000 transfection reagent (Invitrogen, Shanghai, China) according to the manufacturer’s instruction.

### Cell Counting Kit-8 Assay

CCK-8 assay was used to evaluate cell proliferation. Briefly, HepG2 cells were seeded at a density of 4,000 cells per well in 96-well plates and incubated for 1, 2, 3, 4, or 5 days respectively. 10 μl CCK-8 (Dojindo Molecular Technologies, Japan) was added to each well, incubated for 4 h, and mixed gently on an orbital shaker for 2 min before the absorbance value (OD) of each well was measured at 450 nm. Experiments were carried out in triplicate.

### Apoptosis Assay

1 * 10^5^ transfected cells were seeded on 6 cm-diameter plates with RPMI-1640 medium containing 10% FBS for 48 h. Then the cells were harvested, washed twice with ice-cold phosphate-buffered saline (PBS), and re-suspended with binding buffer at a concentration of 1 * 10^6^ cells/ml. The cells were then labeled by using an annexin V-FITC/PI staining kit (eBioscience, United States) according to manufacturer’s instructions. The DNA content of labeled cells was analyzed with fluorescence activated cell sorting (FACS) cytometry (Millipore, United States). Experiments were performed in triplicate.

### Cell Invasion Assay

1 * 10^5^ transfected cells were seeded in 500 μl RPMI-1640 medium on the matrigel in the upper chamber of the Corning® BioCoat™ Matrigel® Invasion Chambers (8 mm pore size; Corning, United States), 750 μl RPMI-1640 medium containing 30% FBS was added in the bottom chamber. The cells were incubated for 24 h at 37°C with 5% CO_2_ and then were fixed in 4% paraformaldehyde and stained with crystal violet solution (Sigma, United States). The cells on the bottom of the membrane were visualized under a microscope and quantified by counting the number of cells in three randomly chosen fields at 100-fold magnification. Experiments were performed in triplicate.

### IHC Staining

IHC staining of BAG2 on 8 μm paraffin sections was performed according to a standard 3,3′-Diaminobenzidine (DAB) staining protocol (Abclone, China) with primary antibody against BAG2 (Abclone, ab115205, 1:200). Two independent pathologists assessed all IHC samples. The extent of cell staining (0–10% positive cells for 0; 11–50% positive cells for 2; 51–80% positive cells for 3; >80% positive cells for 4) and the staining intensity (no staining for 0; slight staining for 1; moderate staining for 2; strong staining for 3) were scored separately and then added to reflect the expression of BAG2 [14].

### Western Blot

Western blotting analysis was conducted as reported in our previous studies [13]. Briefly, transfected cells were cultured for 24 h before total protein was extracted by using RIPA buffer (Beyotime, China) containing proteinase inhibitor cocktail (Roche, Switzerland). The BCA Protein Assay Kit (Beyotime, China) was used to measure the protein concentration, and approximately 30 μg of total protein was separated by 10% SDS-PAGE and transferred onto PVDF membranes (Millipore, United States). The membranes were incubated in 5% skim milk for 1 h at room temperature and then overnight at 4°C with primary antibodies. After the membranes were washed three times, they were incubated with HRP-conjugated secondary antibody. The primary antibodies were rabbit polyclonal anti-BAG2 (Abclonal, China) and rabbit polyclonal anti-GAPDH (Abcam, United States). The secondary antibody was HRP-conjugated anti-rabbit (Sigma, United States).

### Bioinformatics Analysis and Statistical Analysis

Normalized gene expression data (HTSeq-FPKM), phenotype data, survival information, and miRNA expression quantification data of liver hepatocellular carcinoma (LIHC) were downloaded from TCGA through GDC Xena Hub (https://xena.ucsc.edu/public) on March 03, 2020. Progression free survival (PFS) and disease specific survival (DSS) data were downloaded from Kaplan Meier-plotter (http://www.kmplot.com), with the gene expression data of BAG2. The Oncomine database (https://www.oncomine.org/resource/login.html) was used to validate the expression of BAG2 in HCC, with default thresholds: *p*-value < 1E-4, fold change >2 and the gene ranks in the top 10%. GSE64041 (60 pairs of tumor and tumor-adjacent normal samples), GSE25097 (6 liver samples from healthy people, 40 cirrhosis samples, 243 tumor-adjacent normal samples, and 268 tumor samples) and GSE121248 (37 tumor-adjacent normal samples and 70 tumor samples) datasets were downloaded from the Gene Expression Omnibus (GEO) database through R (version 3.6.2), by the GEOquery package. All the data in this part were processed, analyzed and plotted by R (version 3.6.2), and the following packages were used: BiocManager, clusterProfiler, dplyr, ggplot2, ggpubr, limma, org. Hs.eg.db, plyr, survival, survminer, and tidyverse.

The co-expressed genes of BAG2 were constructed into protein-protein interaction (PPI) networks in the STRING database (http://string-db.org). Cytoscape (version 3.7.2) was then used for the visualization of these networks. The hub genes were then identified as the top 10 nodes based on the score generated by the cytoHubba plug-in in Cytoscape (ranked by degree). Gene ontology (GO) and Kyoto Encyclopedia of Genes and Genomes (KEGG) enrichment analysis were conducted by the clusterProfiler package in R (version 3.6.2) [15].

Overall survival (OS), PFS, and DSS of high- and low-BAG2 subgroups of HCC patients were compared using the Kaplan-Meier method with the log-rank test. Cox regression analysis were conducted with SPSS (version 21), to evaluate prognostic factors. The cut-off value of BAG2 was determined by X-tile (version 3.6.1) [16]. Levene’s test was used for homogeneity of variance test. When the variance was assumed even, unpaired *t* test was used to compare the data from two groups and one-factor ANOVA test for multiple groups without post-hoc analysis. Wilcoxon signed rank test was used for the comparison between two groups when the variance was assumed uneven. Correlation analysis between the gene expression of BAG2 and that of the rest genes was conducted by R software (version 3.6.2) with spearman method. The data from *in vitro* experiments were analyzed and plotted by Graphpad Prism 8 (GraphPad Software, La Jolla, CA). All statistical tests were two tailed and *p* value <0.05 was considered significant.

## Results

### BAG2 Was Up-Regulated in HCC

**FIGURE 1:**
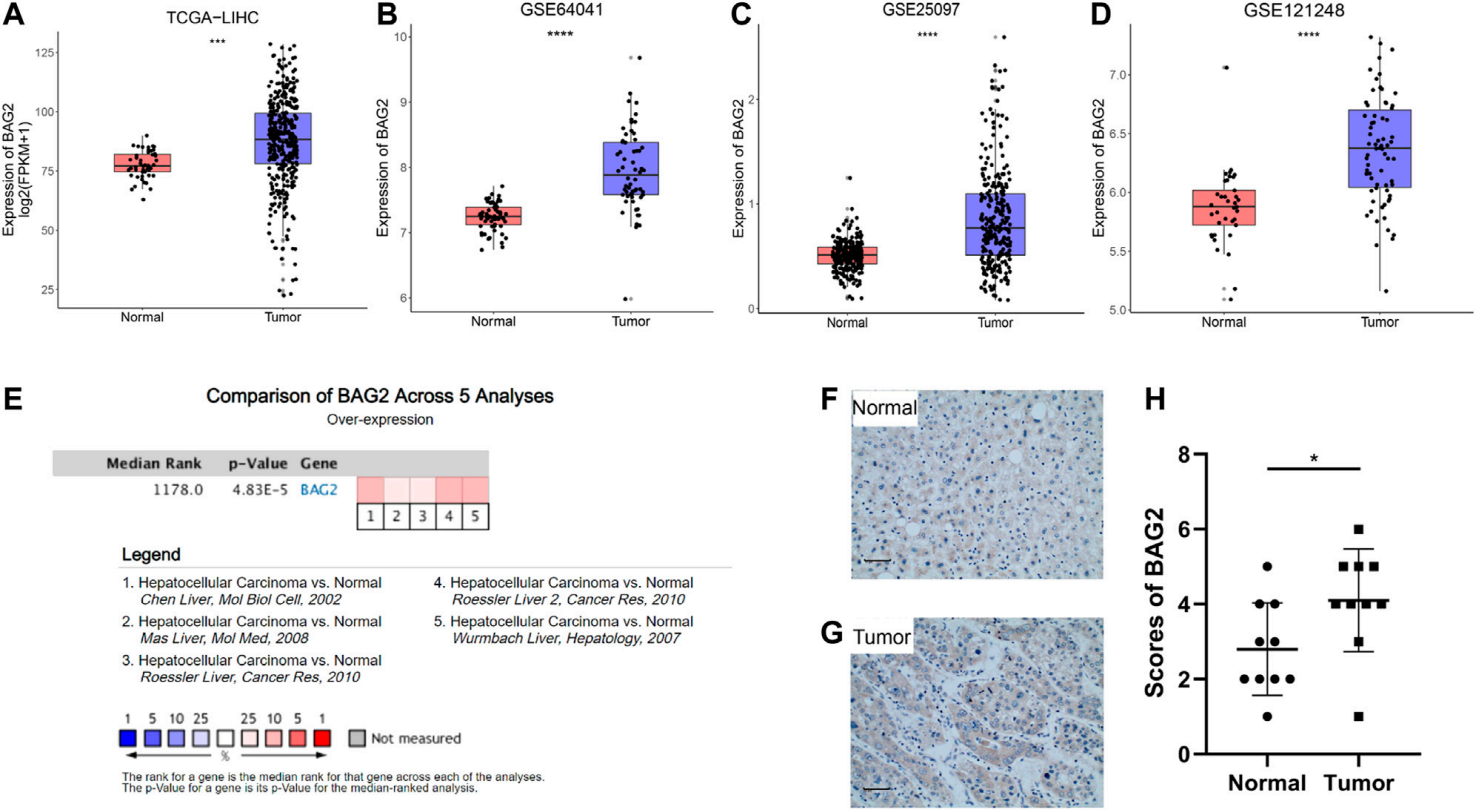
BAG2 was up-regulated in HCC. **(A)** The gene expression of BAG2 was significantly up-regulated in tumor samples (*n* = 374) compared to that in normal livers (*n* = 50), based on bioinformatics analysis of TCGA-LIHC dataset. **(B–D)** Three independent GEO data sets (GSE64041, GSE25097, and GSE121248) showed that BAG2 was overexpressed in HCC tissues when compared with normal tissues. **(E)** Data from the Oncomine database validated that BAG2 was significantly up-regulated in HCC tissues. **(F–H)** Quantification of expression level of BAG2 in hepatocellular carcinoma (*n* = 10) and tumor-adjacent tissue (*n* = 10). *p* < 0.05 was statistically significant, ns: no significant, *p < 0.05, **p < 0.01, ***p < 0.001, ****p < 0.0001.

To delineate the function of BAG2 in HCC, we first examined its expression pattern in the disease. By analyzing the data from the TCGA_LIHC data set, we found that the transcriptional level of BAG2 was considerably increased in tumor tissues when compared with tumor-adjacent normal tissues (Figure 1A, *p* < 0.001). Such an expression pattern was supported by three independent GEO data sets, namely GSE64041, GSE25097 and GSE121248 (Figures 1B–D, *p* < 0.0001). We further validated that BAG2 was significantly up-regulated in HCC by comparing its expression between HCC samples and normal samples across five independent cohorts in the Oncomine database (Figure 1E). In addition, we conducted IHC staining of BAG2 on 10 HCC tissues and 10 tumor-adjacent normal tissues and scored the staining based on the intensity and extent [14]. The results confirmed that the expression of BAG2 was significantly elevated in tumor tissues when compared with normal tissues (Figures 1F–H, *p* < 0.05).

### BAG2 Was Associated With Worse Clinicopathological Features of HCC

**FIGURE 2:**
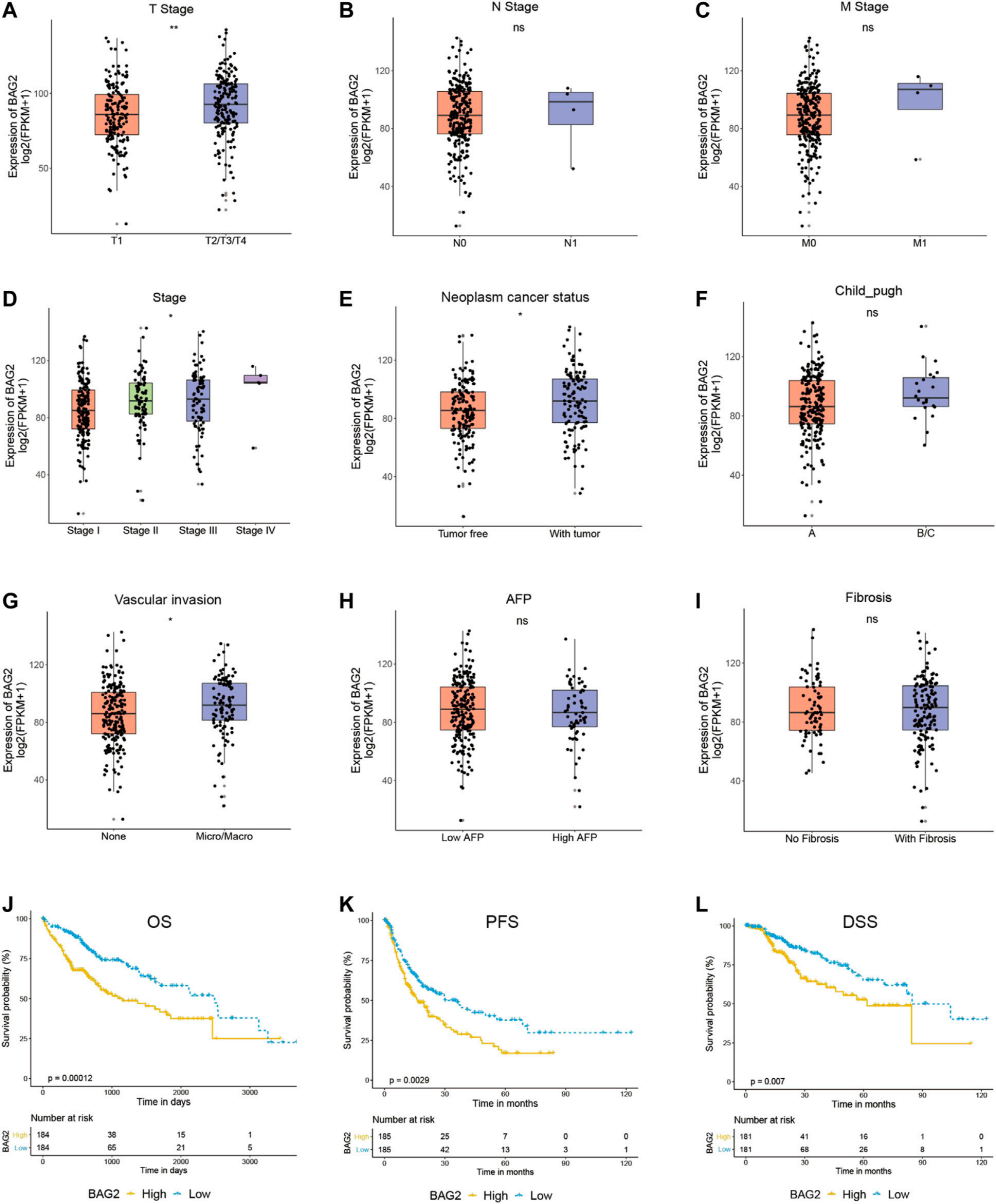
BAG2 was associated with worse clinicopathological features of HCC. **(A–I)** The correlation of the expression of BAG2 with tumor stage **(A)**, lymph node stage **(B)**, metastasis stage **(C)**, disease stage **(D)**, cancer status **(E)**, Child–Pugh classification **(F)**, vascular invasion **(G)**, Fetoprotein (AFP) value **(H)**, and Ishak Fibrosis score **(I)** was analyzed. **(J–L)** High expression of BAG2 was significantly related to poorer overall survival **(J)**, shorter progression free survival **(K)** and shorter disease specific survival (L). *p* < 0.05 was statistically significant, ns: no significant, *p < 0.05, **p < 0.01.

We then examined the relationship between clinicopathological features of HCC patients and the gene expression of BAG2 by manipulating data from the TCGA_LIHC data set. The clinical characteristics of HCC patients were summarized in Supplementary Table S3. A total of 368 cases had gene expression data, survival data and phenotype data. Unavailable or unknown clinical features of the 368 patients were regarded as missing values. Briefly, 44.8% of HCC patients were younger than 60 years old, and more patients were male than female (67.7 vs. 32.3%). Most patients were at relatively good status, because only a small fraction of patients were at T4 stage (3.5%), with lymph node metastasis (N1, 1.1%), with distant metastasis (M1, 0.8%), diagnosed as stage IV (1.1%), or with bad liver function (Child–Pugh classification C, 0.3%). Besides, 56.3% of patients had no vascular invasion, 20.4% had no fibrosis, and 32.1% had no adjacent hepatic tissue inflammation. With respect to cancer status, 33.7% of HCC patients had tumor while 44.0% of them were tumor free. As shown in Figures 2A–I, high level of BAG2 was significantly correlated with advanced T stage (*p* < 0.01) and disease stage (*p* < 0.05). Due to the small number of patients with lymph node or distant metastasis, no correlation was found between N or M stage and BAG2 expression. In addition, patients with poor cancer status (with tumor, *p* < 0.05) or with vascular invasion (*p* < 0.05) was associated with higher expression of BAG2. No correlation was found between the expression of BAG2 and level of AFP, fibrosis or adjacent hepatic tissue inflammation.

As shown in Figures 2J–L, HCC patients with high expression of BAG2 had significantly shorter overall survival (OS, *p* = 0.00012), progression free survival (PFS, *p* = 0.0029), and disease specific survival (DSS, *p* = 0.007). A univariate analysis revealed that low level of BAG2 [hazard ratio (HR): 0.508; 95% confidence interval (CI): 0.357–0.722; *p* < 0.001], good cancer status (HR: 0.372, CI: 0.234–0.590; *p* < 0.001), early T stage (HR: 0.479, CI: 0.334–0.685; *p* < 0.001), without lymph node metastasis (HR: 0.676, CI: 0.469–0.973; *p* = 0.035), and without distant metastasis (HR: 0.613, CI: 0.426–0.883; *p* = 0.009) were all associated with longer OS (Table 1a). We further put these factors into a multivariate Cox regression analysis and found that low BAG2 expression was still an independent factor that correlated to good prognosis (HR: 0.421, CI: 0.247–0.719; *p* = 0.002) (Table 1b).

**TABLE 1:** a. Correlation between clinicopathological characteristics and overall survival and of HCC patients by using Cox regression. b. Multivariate survival analysis after variable selection.

Clinicopathologic variable	HR (95% CI)	*p*-value
a		
BAG2 (low vs. high)	0.508 (0.357–0.722)	<0.001
Inflamation(None vs. Mild/Severe)	0.869 (0.535–1.406)	0.564
Person neoplasm cancer status (tumor free vs. With tumor)	0.372 (0.234–0.590)	<0.001
Age (<60 vs. ≥60)	0.857 (0.603–1.220)	0.393
Race (non-white vs. White)	0.802 (0.559–1.149)	0.229
Gender (male vs. female)	0.801 (0.563–1.141)	0.219
Vascular tumor (None vs. Micro/Macro)	0.758 (0.501–1.147)	0.19
Stage (I vs. II–IV)	0.491 (0.337–0.716)	<0.001
T (T1 vs. T2/T3/T4)	0.479 (0.334–0.685)	<0.001
N (N0 vs. N1/NX)	0.676 (0.469–0.973)	0.035
M (M0 vs. M1/MX)	0.613 (0.426–0.883)	0.009
Fibrosis score (0 vs. 1–6)	1.350 (0.818–2.229)	0.24
AFP (<400 vs. ≥400)	0.944 (0.578–1.542)	0.818
Child-pugh (A vs. B/C)	0.609 (0.300–1.234)	0.168
b		
BAG2 (low vs. high)	0.421 (0.247–0.719)	0.002
Person neoplasm cancer status (tumor free vs. With tumor)	0.566 (0.337–0.951)	0.032
Stage (I vs. II–IV)	0.382 (0.043–3.384)	0.388
T (T1 vs. T2/T3/T4)	1.023 (0.122–8.547)	0.983
N (N0 vs. N1/NX)	0.889 (0.419–1.884)	0.758
M (M0 vs. M1/MX)	1.001 (0.465–2.156)	0.999

### Silencing BAG2 Facilitated Apoptosis, and Repressed Proliferation and Invasion of HCC Cells

**FIGURE 3:**
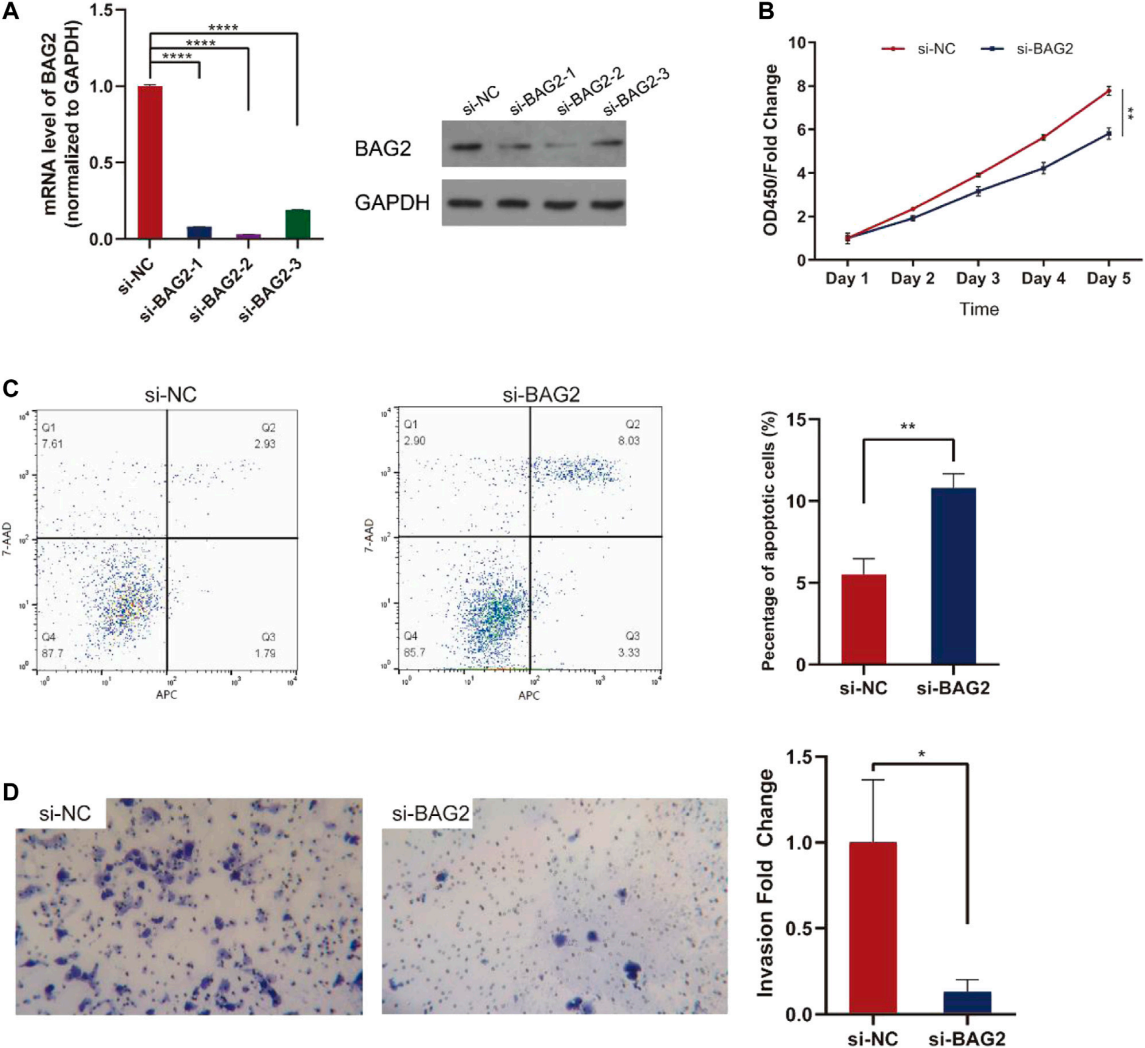
BAG2 suppressed apoptosis, favored proliferation and invasion of HCC. **(A)** Three si-BAG2s dramatically suppressed the expression of BAG2 in HepG2 cells **(B)** CCK-8 assay indicated that HepG2 cells transfected with si-BAG2 had decreased cell proliferation **(C)** Down-regulation of BAG2 in HepG2 cells led to increased percentage of apoptotic cells **(D)** Down-regulation of BAG2 in HepG2 cells impaired the invasion capability of tumor cells. All values shown were mean ± SD. *p* < 0.05 was statistically significant, *p < 0.05, **p < 0.01, ***p < 0.001, ****p < 0.0001.

To investigate how BAG2 affected biological activities of HCC, we transfected HepG2 cells with three si-BAG2s or si-NC. All the three si-BAG2s dramatically down-regulated the expression of BAG2 on the mRNA and protein level, and si-BAG2-2 had the strongest inhibition (Figure 3A). We then used si-BAG2-2 for further experiments. As shown in Figure 3B, silencing the expression of BAG2 in HCC cells significantly impaired cell proliferation on day 4 (*p* < 0.01) and day 5 (*p* < 0.01). Besides, HCC cells with down-regulated expression of BAG2 showed a significantly increased percentage of apoptotic cells (Figure 3C, *p* < 0.01). Because Lisha Sun et al. reported that BAG2 promoted metastasis of gastric cancer [7], we further conducted invasion assay and the result suggested that BAG2 knockdown dramatically decreased the amount of cells invaded though the matrigel (Figure 3D).

### BAG2 Might Regulate Ribosome Biogenesis

**FIGURE 4:**
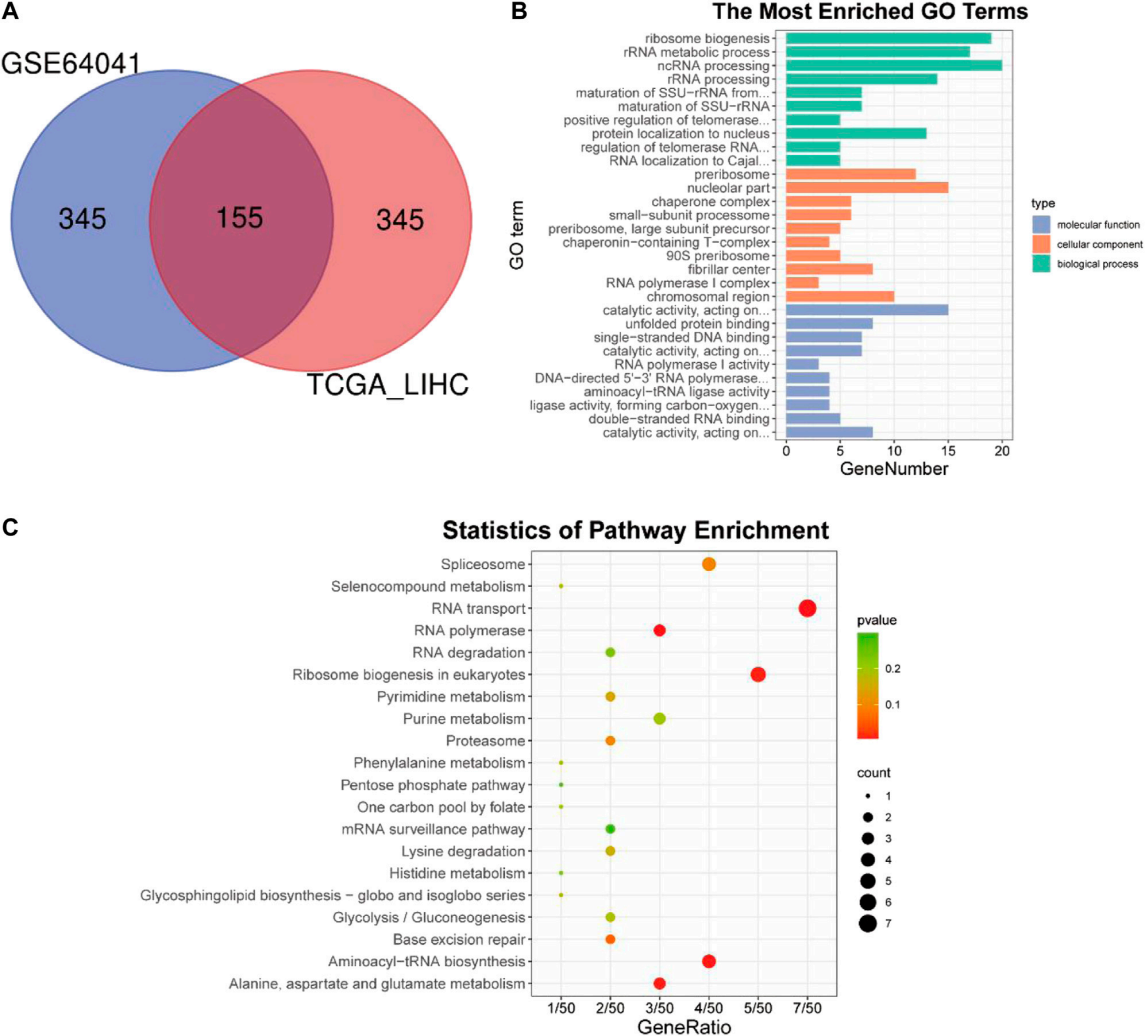
Co-expression analysis and subsequent GO and KEGG enrichment analysis of BAG2. **(A)** Co-expression analysis of BAG2 in TCGA_LIHC and GSE64041 data sets. **(B)** GO enrichment analysis of the common co-expressed genes of BAG2. **(C)** KEGG enrichment analysis of the common co-expressed genes of BAG2.

The above analysis and experiments indicated that BAG2 was up-regulated in HCC and might favor the progression of the disease; we then intended to understand the underlying regulatory mechanisms. Correlation analysis was conducted independently between the expression of BAG2 and that of other genes in tumor samples of the TCGA_LIHC and GSE64041 data sets. 10,918 genes and 3,837 genes were identified as co-expressed genes in the TCGA_LIHC and GSE64041 data sets respectively (*p* value cutoff = 0.05). Based on the correlation coefficient, the top 500 co-expressed genes from the two data sets were intersected and 155 genes were found to be the common co-expressed genes for BAG2 (Figure 4A; Supplementary Table S4). These co-expressed genes were then undergoing GO and KEGG analysis. The significantly affected biological processes (BP) were “RNA biogenesis and processing like ribosome biogenesis,” “ribosome RNA (rRNA) metabolic process,” “non-coding RNA (ncRNA) processing,” “rRNA processing,” “maturation of SSU-rRNA from tricistronic rRNA transcript (SSU-rRNA, 5.8S rRNA, LSU-rRNA),” and “positive regulation of telomerase RNA localization to Cajal body” (Figure 4B). The significantly enriched molecular functions (MF) of these co-expressed genes were “catalytic activity,” “unfolded protein binding,” “RNA polymerase activity,” and “aminoacyl-tRNA ligase activity” (Figure 4B). According to the KEGG analysis, “RNA transport,” “RNA polymerase,” “aminoacyl-tRNA biosynthesis,” “Alanine, aspartate and glutamate metabolism,” and “ribosome biogenesis in eukaryotes” were significantly enriched terms (Figure 4C; Table 2).

**TABLE 2:** KEGG analysis of the co-expressed genes of BAG2 in HCC.

ID	Description	*p* value
hsa03013	RNA transport	0.004534547
hsa03020	RNA polymerase	0.005399698
hsa00970	Aminoacyl-tRNA biosynthesis	0.007045845
hsa00250	Alanine, aspartate and glutamate metabolism	0.008232431
hsa03008	Ribosome biogenesis in eukaryotes	0.009074458

### PPI Network Construction and Analysis of Hub Genes

**FIGURE 5:**
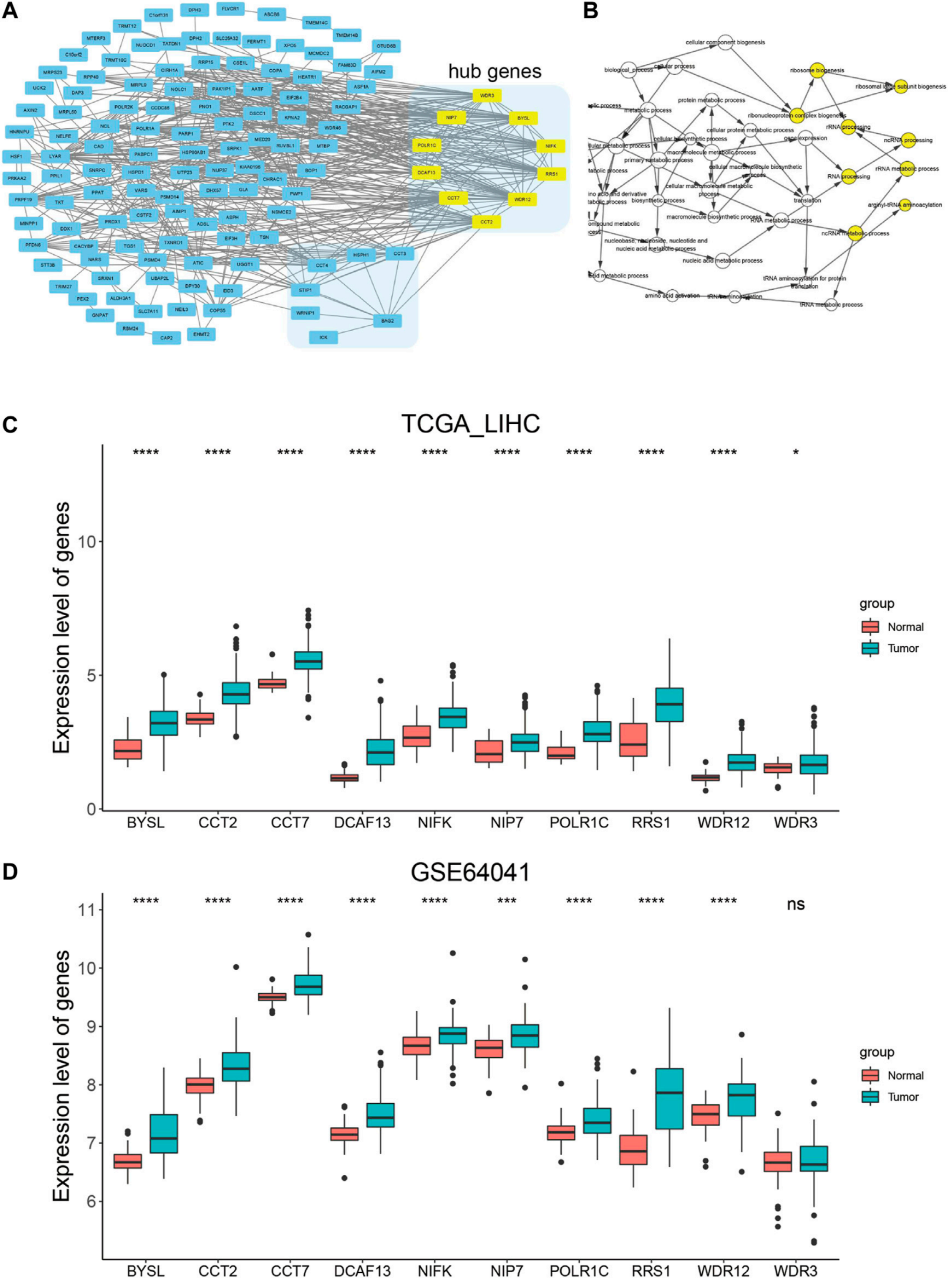
PPI network construction and analysis of hub genes. **(A)** PPI network construction of the co-expressed genes of BAG2 and identification of hub genes (yellow block). **(B)** Biological process analysis of hub genes by using the BINGO plug-in in the Cytoscape software. **(C,D)** Expression analysis of the 10 hub genes in tumor and non-tumor samples in TCGA_LIHC **(C)** and GSE64041 **(D)** data sets. *p* < 0.05 was statistically significant, *p < 0.05, **p < 0.01, ***p < 0.001, ****p < 0.0001.

**FIGURE 6:**
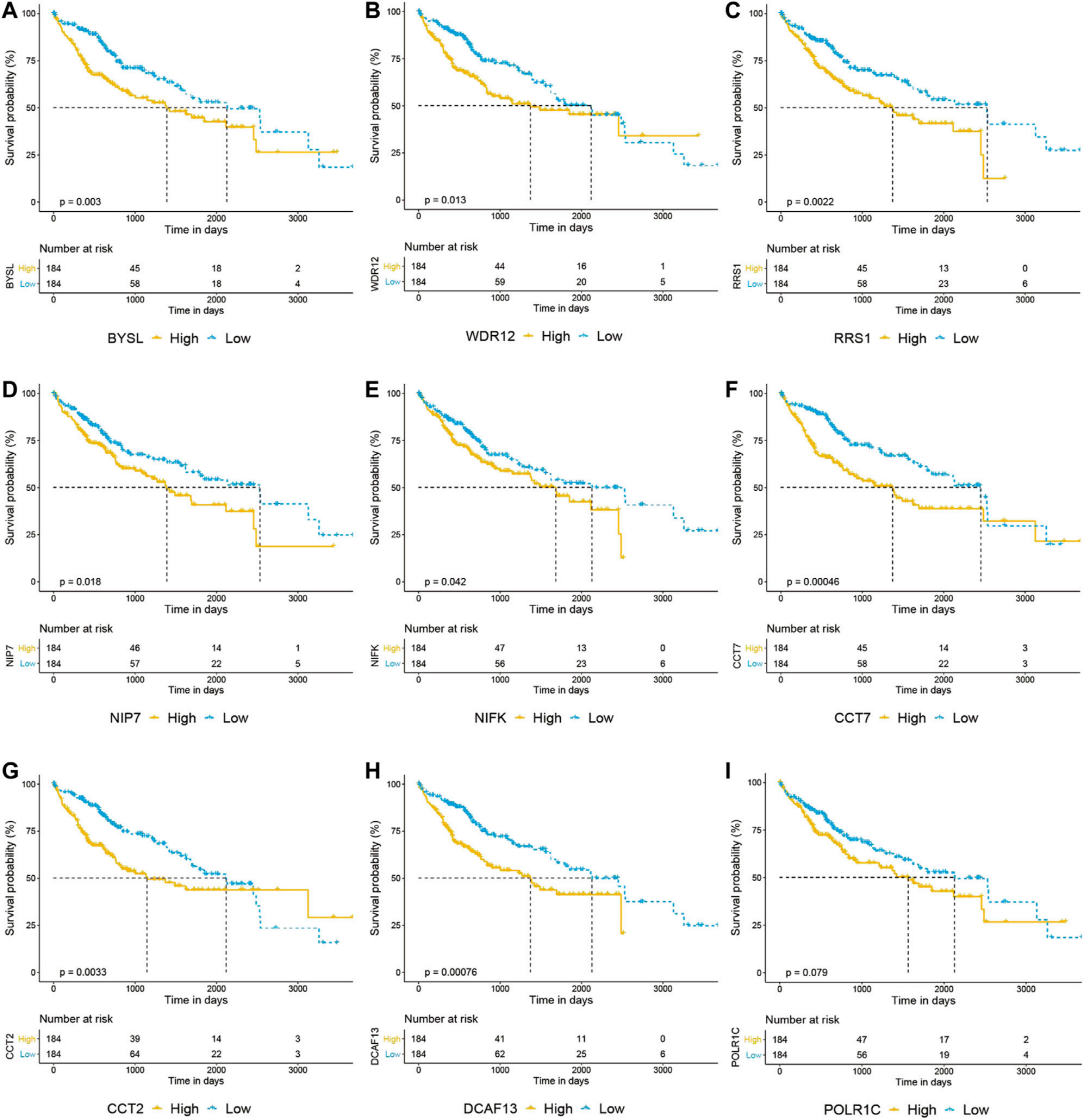
Overall survival analysis of hub genes in HCC. **(A–I)** Prognostic analysis of BYSL, WDR12, RRS1, NIP7, NIFK, CCT7, CCT2 and DCAF13 indicated that high expression of these genes predicted unfavorable prognosis in HCC **(A–H)**, while no significant correlation was observed between the expression of POLR1C and overall survival of HCC patients **(I)**.

We then uploaded BAG2 and its 155 co-expressed genes into the STRING database and constructed a BAG2-contained PPI network, which was visualized by the Cytoscape software (Figure 5A). By using the cytoHubba plug-in, the 10 hub genes (ranked by degree) were identified to be WD repeat domain 12 (WDR12), bystin like (BYSL), DDB1 and CUL4 associated factor 13 (DCAF13), WD repeat domain 3 (WDR3), chaperonin containing TCP1 subunit 2 (CCT2), ribosome biogenesis regulator 1 homolog (RRS1), nucleolar pre-RRNA processing protein NIP7 (NIP7), RNA polymerase I and III subunit C (POLR1C), nucleolar protein interacting with the FHA domain of MKI67 (NIFK), chaperonin containing TCP1 subunit 7 (CCT7) (Figure 5A, yellow blocks; Supplementary Table S4). The biological process analysis of these hub genes was conducted by using the BINGO plug-in the Cytoscape software. “Ribosome biogenesis,” “ribosomal large subunit biogenesis,” “ribonucleoprotein complex biogenesis,” “rRNA processing,” “ncRNA processing,” “rRNA metabolic process,” “RNA processing,” “arginyl-tRNA aminoacylation” and “ncRNA metabolic process” were significantly altered, consistent with the GO and KEGG enrichment analysis of the co-expressed genes of BAG2 (yellow modules, Figure 5B). Except WDR3, all the rest nine hub genes were significantly up-regulated in tumors when compared to tumor-adjacent normal tissues in both the TCGA_LIHC and GE64041 data sets (Figures 5C,D). We then analyzed the prognostic significance of the nine hub genes in the TCGA_LIHC data set, and the result suggested that WDR12, BYSL, DCAF13, CCT2, RRS1, NIP7, NIFK, and CCT7 at high expression levels were all associated with significantly unfavorable prognosis in HCC (Figures 6A–H), while no significant correlation was observed between the expression of POLR1C and OS of HCC patients (Figure 6I). In addition, previous studies revealed that most of these hub genes were required or inducible for ribosome biogenesis [17–23]. Further, we detected the transcriptional level of the 10 hub genes in HepG2 cells with BAG2 silencing, and found that all of them showed a significant down-regulation after BAG2 knockdown, suggesting BAG2 might affect ribosome biogenesis through these genes (Supplementary Figure S1).

## Discussion

Identifying oncogenes and their related regulatory mechanisms in tumors is of great help in developing new potential therapies. For instance, cetuximab targeting epidermal growth factor receptor (EGFR), bevacizumab against vascular endothelial growth factor receptor (VEGFR), and dabrafenib inhibiting BRAF V600E mutation has become cornerstone of anti-tumor treatments and considerably improve the prognosis of advanced cancer patients [24–26].

The BAG family, which is evolutionarily conserved, might serve as a new potential therapeutic target in cancer treatment since studies show that members of this family are related to chemo-resistance, invasion and apoptosis of tumors [27–31]. For instance, Tugba Kizilboga et al. reported that BAG1 favored survival of breast cancer cells by phosphorylating the pro-apoptotic Bad protein through activation of the Akt and Raf kinase pathways [30]. Anja Bruchmann et al. found that BAG5 was over-expressed in prostate cancer and played a role in preventing stress-induced cell death [31]. Jiling Lv et al. showed that down-regulation of BAG1 could sensitize non-small cell lung cancer (NSCLC) cells to chemotherapy like cisplatin [28].

Some studies also investigated the role of BAG family in HCC. Wenkai Ni et al. reported that BAG1 was over-expressed in the nucleus of HCC cells and high expression of the gene increased resistance of HCC cells to doxorubicin [32]. The role of BAG3 in HCC is somewhat controversial, since Dehui Kong et al. showed that high expression of BAG3 suppressed cell proliferation while Heng Xiao et al. reported that BAG3 elevation favored invasiveness and angiogenesis in HCC [33, 34]. In this study, we for the first time, to our knowledge, investigated the role of BAG2 in HCC. Bioinformatics analysis of the data from TCGA and GEO data sets, and IHC staining of BAG2 on collected tumor tissues and tumor-adjacent normal tissues all showed that BAG2 was significantly up-regulated in HCC when compared with normal liver samples. A similar expression pattern of BAG1 and BAG3 has been reported in HCC, indicating that the BAG family might actively involve in the development of the disease [32, 33]. Elevated expression of BAG2 was further found to be associated with worse clinicopathological features. *In vitro* experiments showed that BAG2 might play a role in many biological behaviors of HCC, like favoring cell proliferation, repressing apoptosis and favoring tumor invasion (Figures 3A–E). With these pro-tumor effects in HCC, BAG2 at higher levels was found to be related to shorter survival of HCC patients and could serve as an independent prognostic marker (Figures 2J–L; Table 2). Other studies also revealed that BAG2 supported proliferation and/or metastasis of tumors like breast cancer and oral cancer [7, 9, 10]. Taken together, BAG2 might act as an oncogene across a set of tumors.

To understand the underlying mechanism of BAG2 in favoring proliferation and invasion of HCC, we conducted co-expression analysis in the TCGA_LIHC and GSE64041 data sets (Figure 4A). GO and KEGG enrichment analysis showed that BAG2 might regulate ribosome biogenesis of HCC (Figures 4B,C). Indeed, previous studies found that BYSL, NIP7, DCAF13, WDR12, NIFK, and RRS1 regulated ribosome biogenesis [17–23]. These genes were found to be hub genes of BAG2, and they were down-regulated in HCC cells after silencing of BAG2 (Supplementary Figure S1). Ribosome biogenesis is an old topic, but gains accumulating attention in cancers in recent years [2, 35]. Researchers find that human pathological conditions characterized by an up-regulated ribosome biogenesis are at an increased risk of cancer onset, and ribosome biogenesis not only supports cell proliferation but facilitates tumor invasion [2, 3, 36]. P53 plays an important role in balancing ribosome biogenesis rate and cell cycle progression [36]. Studies showed that p53 negatively controlled ribosome biogenesis, by inhibiting transcription of both nuclear RNA polymerases I and III, two key determinants for the ribosome production [37, 38]. However, cancer cells harboring p53 mutation might lose its negative control on ribosome biogenesis [36]. Since BAG2 contributes to the accumulation of mutant p53, which could interact with c-Myc and enhance c-Myc-dependent rDNA transcription for ribosome biogenesis, mutant p53 might be a downstream mediator of BAG2 in regulating the biogenesis [8, 39]. It is worthy to point out that mutant p53 occurs frequently in HCC and related to poor differentiation and vascular invasion [40, 41]. Further studies are required to determine whether BAG2 plays a predominant role in the accumulation of mutant p53 protein. In addition, PI3K/Akt/mTOR and MAPK signaling pathways might be other potential downstream mediators of BAG2 in regulating ribosome biogenesis, as these pathways were reported to regulate ribosome biogenesis and could be modulated by other members of the BAG family [27, 29, 30, 42].

Besides, it is possible that BAG2 regulates apoptosis, proliferation and invasion via mechanisms other than ribosome biogenesis regulation, since BAG proteins could functionally interact with a variety of binding partners including Hsp70 [5, 43]. Indeed, the BAG2-Hsp70 complex plays an essential role in the protein homeostasis [43]. Studies found that Hsp70 suppressed apoptosis through multiple pathways, like preventing Bax translocation, preventing oligomerization of apoptotic protease activating factor 1 (APAF1), neutralizing the apoptosis-inducing factor (AIF) and inhibiting the activation of Caspase-3 [44–48]. Moreover, the BAG family is well known for its capability in preventing cell death through interaction with Bcl-2, which plays a prominent role in suppressing apoptosis and enhancing cell survival in response to diverse apoptotic stimuli [5, 49]. Thus, BAG2 might inhibit cell apoptosis in HCC via enhancing the anti-apoptotic function of Bcl-2 [50]. Lisha Sun et al. reported that BAG2 could bind to ERK1/2 in gastric cancer, and promote proliferation and metastasis of the disease [7]. Numerous studies showed that ERK1/2 promoted progression of HCC, thus the signaling might be an important downstream pathway of BAG2 in the disease [51–53]. In addition, BAG2 could interact with pro-peptide region of pro-cathepsin B, block the auto-cleavage process and facilitate the secretion of the enzyme, which induces metastasis of TNBC [9]. Since cathepsin B participates in the progression of HCC, it would be of great importance to investigate the BAG2/cathepsin B axis in HCC by future studies [54, 55].

## Conclusion

In summary, this work showed that BAG2 was up-regulated in HCC and high expression of the gene correlated to unfavorable prognosis in the disease. BAG2 might favor cell proliferation, repress apoptosis and facilitate tumor invasion in HCC, probably through regulation on ribosome biogenesis via genes like WDR12, BYSL, and DCAF13 [17, 19, 20].

## Data Availability

The original contributions presented in the study are included in the article/Supplementary Material, further inquiries can be directed to the corresponding author.
