# Supplementary material for: Bcl-2 Associated Athanogene 2 (BAG2) is Associated With Progression and Prognosis of Hepatocellular Carcinoma: A Bioinformatics-Based Analysis
Source: Pathol Oncol Res. 2021 Apr 1;27:594649. doi: 10.3389/pore.2021.594649 (PMC8262200; doi:10.3389/pore.2021.594649)

Supplement table 1: Clinical characteristics of 10 HCC patients from the Third Xiangya Hospital

| Demographic data | | Clinical data | | | | | | Pathological data | | | |
| --- | --- | --- | --- | --- | --- | --- | --- | --- | --- | --- | --- |
| Gender | Age | Stage | HBV | HCV | liver cirrhosis | Child-pugh | Grade | Hepatocyte | Glypican-3 | Ki67 | MVI |
| M | 37 | IIIB | Yes | No | Yes | A | II | ++ | - | 40% | M2 |
| M | 43 | II | Yes | No | Yes | A | III | - | + | 50% | M2 |
| F | 54 | II | Yes | No | Yes | A | II | +++ | +++ | 50% | M0 |
| M | 60 | I | Yes | No | No | A | II | +++ | - | 10% | M0 |
| M | 55 | IIIB | Yes | No | Yes | A | II | + | + | 40% | M1 |
| F | 48 | I | Yes | No | Yes | A | II | + | + | 20% | M1 |
| M | 37 | IIIA | Yes | No | Yes | A | III | ++ | +++ | 40% | M2 |
| F | 76 | I | Yes | No | No | A | II | ++ | - | 20% | M0 |
| M | 63 | I | Yes | No | Yes | A | II | + | ++ | 40% | M0 |
| M | 73 | I | Yes | No | Yes | A | II | + | + | 60% | M0 |

Supplement table 2: Primers of genes

| Symbol | Forward | Reverse |
| --- | --- | --- |
| WDR12 | TGGCTTGGGTGAAAAAAGACA | TCCCATAAGAGAATAGTCTGGTCCAT |
| BYSL | AGAAGGCTGCCACAATGACA | GACATGACTGTCTCAACCTC |
| CCT2 | CTCTTGTCACAGGTGGTGAAATT | CTCAGAACAGCCTCCTCCATAA |
| WDR3 | ATGCCTCGAGATATGGGGCTCACCAAGCAGTA | ATGCAAGCTTGTCGACTGCAAGGTTTACAAC |
| DCAF13 | TTTCCTGTAGACAAAAGTCGAAGCA | GCATTAGCTTTCCACAGGCG |
| RRS1 | CCCTACCGGACACCAGAGTAA | CCGAAAAGGGGTTGAAACTTCC |
| NIP7 | CCGGGTGTACTATGTGAGTGAGAA | TTGTGGGTTTTAGTGAATTTTCCA |
| CCT7 | GATTGGAGGCGAGAGGTACAATT | CCACTGAATCATTCTTGATGGCC |
| POLR1C | CGGTTATGATGATGCCTGGGA | TGCGTCAATTCCCACCATGT |
| NIFK | AGGTGGCGCAGGTTCGCAAG | TGGTGTGGGGCCCTGGCTATC |
| GAPDH | CCATGACAACTTTGGTATCGTGGAA | GGCCATCACGCCACAGTTTC |
| BAG2 | AAACCCCCAGCAGCAAGAAT | GCACCTCAGATGAACATGCAC |

Supplement table 3: TCGA liver hepatocellular carcinoma patient characteristics.

| Clinical characteristics | | Total (n=368) | % |
| --- | --- | --- | --- |
| Age |  |  |  |
|  | <60 | 165 | 44.8 |
|  | ≥60 | 203 | 55.2 |
| T |  |  |  |
|  | T1 | 182 | 49.5 |
|  | T2 | 92 | 25.0 |
|  | T3 | 78 | 21.2 |
|  | T4 | 13 | 3.5 |
|  | Tx | 3 | 0.6 |
| N |  |  |  |
|  | N0 | 250 | 67.9 |
|  | N1 | 4 | 1.1 |
|  | Nx | 114 | 31.0 |
| M |  |  |  |
|  | M0 | 265 | 72.0 |
|  | M1 | 3 | 0.8 |
|  | Mx | 100 | 27.2 |
| Stage |  |  |  |
|  | I | 172 | 46.7 |
|  | II | 85 | 23.1 |
|  | III | 83 | 22.6 |
|  | IV | 4 | 1.1 |
|  | NA | 24 | 6.5 |
| Person neoplasm cancer status |  |  |  |
|  | With tumor | 124 | 33.7 |
|  | Tumor free | 162 | 44.0 |
|  | NA | 82 | 22.3 |
| Adjacent hepatic tissue inflammation extent |  |  |  |
|  | None | 118 | 32.1 |
|  | Mild | 100 | 27.2 |
|  | Severe | 17 | 4.6 |
|  | NA | 133 | 36.1 |
| Vascular invasion |  |  |  |
|  | None | 207 | 56.3 |
|  | Micro | 91 | 24.7 |
|  | Macro | 16 | 4.3 |
|  | NA | 54 | 14.7 |
| Gender |  |  |  |
|  | Female | 119 | 32.3 |
|  | Male | 249 | 67.7 |
| Ishak Fibrosis score |  |  |  |
|  | No Fibrosis | 75 | 20.4 |
|  | Portal Fibrosis | 31 | 8.4 |
|  | Fibrous Speta | 27 | 7.3 |
|  | Nodular Formation and Incomplete Cirrhosis | 9 | 2.4 |
|  | Established Cirrhosis | 70 | 19.0 |
|  | NA | 156 | 42.4 |
| Race |  |  |  |
|  | Not White | 175 | 47.6 |
|  | White | 183 | 49.7 |
|  | NA | 10 | 2.7 |
| Fetoprotein (AFP) value |  |  |  |
|  | ≥400 | 63 | 17.1 |
|  | <400 | 215 | 58.4 |
|  | NA | 90 | 24.5 |
| Child–Pugh classification |  |  |  |
|  | A | 218 | 59.2 |
|  | B | 21 | 5.7 |
|  | C | 1 | 0.3 |
|  | NA | 128 | 34.8 |

Supplement table 4: the common co-expressed genes of BAG2 in TCGA_LIHC and GSE64041 data sets.

| Symbol | TCGA_LIHC correlation | TCGA_LIHC p-value | GSE64041 correlation | GSE64041 p-value |
| --- | --- | --- | --- | --- |
| BYSL | 0.532119966 | 9.93E-29 | 0.604550559 | 3.13E-07 |
| DCAF13 | 0.49021716 | 5.25E-24 | 0.634347363 | 5.27E-08 |
| WDR12 | 0.467421287 | 1.07E-21 | 0.503460112 | 4.13E-05 |
| CCT2 | 0.46427168 | 2.16E-21 | 0.509177734 | 3.26E-05 |
| NIP7 | 0.424990959 | 7.79E-18 | 0.479484223 | 0.000106108 |
| NIFK | 0.404440378 | 3.76E-16 | 0.454397134 | 0.000265279 |
| RRS1 | 0.383370004 | 1.53E-14 | 0.697543945 | 5.91E-10 |
| WDR3 | 0.374830664 | 6.39E-14 | 0.468214153 | 0.000161558 |
| POLR1C | 0.374324141 | 6.95E-14 | 0.44864239 | 0.00032415 |
| CCT7 | 0.421424192 | 1.56E-17 | 0.459554808 | 0.000220991 |
| PWP1 | 0.371855386 | 1.04E-13 | 0.458830628 | 0.000226772 |
| TKT | 0.598720973 | 9.55E-38 | 0.455372671 | 0.000256328 |
| TXNRD1 | 0.590440288 | 1.65E-36 | 0.507014929 | 3.56E-05 |
| PAK1IP1 | 0.55824146 | 5.03E-32 | 0.455511924 | 0.000255072 |
| ABCB6 | 0.549361125 | 7.15E-31 | 0.509949045 | 3.16E-05 |
| HSP90AB1 | 0.538749127 | 1.54E-29 | 0.616536177 | 1.56E-07 |
| PRDX1 | 0.514572313 | 1.13E-26 | 0.450827045 | 0.000300527 |
| TRIM16 | 0.513035722 | 1.70E-26 | 0.45789906 | 0.000234411 |
| SRXN1 | 0.509656558 | 4.08E-26 | 0.539989733 | 8.47E-06 |
| RBM24 | 0.502416984 | 2.58E-25 | 0.553894801 | 4.41E-06 |
| PPIL1 | 0.492247433 | 3.21E-24 | 0.532791016 | 1.17E-05 |
| NMRAL2P | 0.491925799 | 3.47E-24 | 0.496689161 | 5.43E-05 |
| HSPD1 | 0.490081593 | 5.43E-24 | 0.589572956 | 7.18E-07 |
| NUDCD1 | 0.487558877 | 9.97E-24 | 0.581360096 | 1.11E-06 |
| SRPK1 | 0.479659803 | 6.47E-23 | 0.549284709 | 5.50E-06 |
| LYAR | 0.479577942 | 6.60E-23 | 0.431558536 | 0.000575817 |
| TMEM14B | 0.478459434 | 8.57E-23 | 0.486798006 | 8.01E-05 |
| SPATA5 | 0.477986098 | 9.56E-23 | 0.550054278 | 5.30E-06 |
| XPO5 | 0.470290544 | 5.60E-22 | 0.499144776 | 4.92E-05 |
| TMEM14C | 0.468377031 | 8.63E-22 | 0.457568393 | 0.000237179 |
| CCT3 | 0.463341834 | 2.66E-21 | 0.590307077 | 6.90E-07 |
| TRIM27 | 0.462603849 | 3.13E-21 | 0.522668234 | 1.84E-05 |
| RPP40 | 0.461700176 | 3.82E-21 | 0.453809136 | 0.000270812 |
| PSMD14 | 0.461049941 | 4.41E-21 | 0.446469595 | 0.000349312 |
| NEIL3 | 0.460059428 | 5.48E-21 | 0.551845688 | 4.87E-06 |
| MCMDC2 | 0.45988548 | 5.69E-21 | 0.574366565 | 1.60E-06 |
| CPLX2 | 0.457992382 | 8.60E-21 | 0.518102192 | 2.24E-05 |
| CDC123 | 0.457826224 | 8.92E-21 | 0.516349419 | 2.41E-05 |
| SNRPC | 0.457425708 | 9.73E-21 | 0.519239724 | 2.13E-05 |
| CAP2 | 0.445917876 | 1.13E-19 | 0.582549952 | 1.04E-06 |
| CCT4 | 0.443858637 | 1.74E-19 | 0.544039851 | 7.03E-06 |
| DPH2 | 0.442979854 | 2.09E-19 | 0.452961151 | 0.000278976 |
| ASF1A | 0.442321801 | 2.39E-19 | 0.463578259 | 0.000191255 |
| NUP37 | 0.441456574 | 2.86E-19 | 0.480993999 | 0.000100185 |
| ASPH | 0.441329657 | 2.94E-19 | 0.459016924 | 0.000225272 |
| NSMCE2 | 0.439227853 | 4.52E-19 | 0.495140665 | 5.77E-05 |
| AIFM2 | 0.437881587 | 5.95E-19 | 0.435569968 | 0.000504497 |
| WASHC5 | 0.43723856 | 6.79E-19 | 0.491198853 | 6.75E-05 |
| MRPS23 | 0.433765484 | 1.37E-18 | 0.505972745 | 3.72E-05 |
| CACYBP | 0.433488664 | 1.45E-18 | 0.539680496 | 8.60E-06 |
| VARS1 | 0.432009105 | 1.95E-18 | 0.505062296 | 3.86E-05 |
| KPNA2 | 0.431465082 | 2.17E-18 | 0.431617884 | 0.000574698 |
| UTP4 | 0.429958762 | 2.93E-18 | 0.452228283 | 0.000286213 |
| SLC7A11 | 0.428928405 | 3.59E-18 | 0.560452404 | 3.21E-06 |
| CCDC86 | 0.427449307 | 4.81E-18 | 0.462818415 | 0.000196574 |
| DDX1 | 0.426970977 | 5.28E-18 | 0.442673247 | 0.000397579 |
| PNO1 | 0.425187241 | 7.50E-18 | 0.524686169 | 1.68E-05 |
| CCDC77 | 0.42353015 | 1.04E-17 | 0.534878304 | 1.07E-05 |
| RUVBL1 | 0.422491478 | 1.27E-17 | 0.480116068 | 0.000103591 |
| ATP6V1C1 | 0.421746455 | 1.46E-17 | 0.441122115 | 0.000418989 |
| UCK2 | 0.420155249 | 1.99E-17 | 0.630300205 | 6.79E-08 |
| SLC25A32 | 0.417791084 | 3.12E-17 | 0.501910919 | 4.39E-05 |
| CSTF2 | 0.414728625 | 5.58E-17 | 0.45660865 | 0.000245382 |
| NCL | 0.413036437 | 7.68E-17 | 0.454405473 | 0.000265202 |
| TATDN1 | 0.410937289 | 1.14E-16 | 0.454826372 | 0.000261306 |
| STIP1 | 0.410093638 | 1.33E-16 | 0.542320319 | 7.61E-06 |
| RRP15 | 0.406006299 | 2.83E-16 | 0.60355622 | 3.32E-07 |
| MED20 | 0.405747601 | 2.96E-16 | 0.609869877 | 2.31E-07 |
| MINPP1 | 0.404202826 | 3.93E-16 | 0.44486324 | 0.000369043 |
| WDCP | 0.402751764 | 5.11E-16 | 0.474818347 | 0.000126505 |
| C1orf131 | 0.402573507 | 5.28E-16 | 0.600423106 | 3.95E-07 |
| ICK | 0.402412905 | 5.44E-16 | 0.542728549 | 7.47E-06 |
| MTDH | 0.402412401 | 5.44E-16 | 0.46895784 | 0.000157208 |
| OTUD6B | 0.402268783 | 5.58E-16 | 0.483610816 | 9.06E-05 |
| BZW2 | 0.401816024 | 6.06E-16 | 0.566457091 | 2.39E-06 |
| TSN | 0.399275927 | 9.57E-16 | 0.570742489 | 1.92E-06 |
| ATIC | 0.398873686 | 1.03E-15 | 0.481273643 | 9.91E-05 |
| EIF3H | 0.397520522 | 1.31E-15 | 0.522976883 | 1.81E-05 |
| UGGT1 | 0.397492592 | 1.32E-15 | 0.535065198 | 1.06E-05 |
| TRIM54 | 0.396039583 | 1.70E-15 | 0.518827494 | 2.17E-05 |
| MRPL50 | 0.395523957 | 1.87E-15 | 0.430372295 | 0.000598592 |
| SLC22A11 | 0.395361877 | 1.92E-15 | 0.482998148 | 9.28E-05 |
| DSCC1 | 0.395109039 | 2.01E-15 | 0.460195048 | 0.000215993 |
| AARS1 | 0.394626722 | 2.19E-15 | 0.432418181 | 0.000559805 |
| ALDH3A1 | 0.393833458 | 2.51E-15 | 0.502447031 | 4.30E-05 |
| EIF2B4 | 0.393193311 | 2.81E-15 | 0.517325224 | 2.31E-05 |
| PPAT | 0.393135909 | 2.84E-15 | 0.450887337 | 0.000299898 |
| VIRMA | 0.388882438 | 5.96E-15 | 0.588348356 | 7.67E-07 |
| NOLC1 | 0.388822461 | 6.02E-15 | 0.515343736 | 2.51E-05 |
| TRMT10C | 0.388693469 | 6.16E-15 | 0.459449492 | 0.000221823 |
| WDR46 | 0.388290629 | 6.60E-15 | 0.62842038 | 7.63E-08 |
| TOX3 | 0.386453129 | 9.06E-15 | 0.559384158 | 3.38E-06 |
| HSPH1 | 0.383795327 | 1.43E-14 | 0.581765126 | 1.09E-06 |
| AIMP1 | 0.382700163 | 1.72E-14 | 0.561138827 | 3.10E-06 |
| FERMT1 | 0.382125937 | 1.89E-14 | 0.672904411 | 3.87E-09 |
| PRKAA2 | 0.382004335 | 1.93E-14 | 0.579594214 | 1.22E-06 |
| TWNK | 0.380768832 | 2.38E-14 | 0.615853658 | 1.63E-07 |
| CAD | 0.380479525 | 2.50E-14 | 0.520696662 | 2.00E-05 |
| RACGAP1 | 0.379807434 | 2.79E-14 | 0.426614156 | 0.000676242 |
| SUV39H2 | 0.378639546 | 3.40E-14 | 0.527585956 | 1.48E-05 |
| PFDN6 | 0.378324889 | 3.58E-14 | 0.583119311 | 1.01E-06 |
| APIP | 0.376467735 | 4.88E-14 | 0.434999459 | 0.000514125 |
| POLR2K | 0.374574152 | 6.67E-14 | 0.487239354 | 7.88E-05 |
| UTP23 | 0.372631676 | 9.17E-14 | 0.619468401 | 1.31E-07 |
| DPH3 | 0.37221938 | 9.81E-14 | 0.451515487 | 0.000293414 |
| RAP1GDS1 | 0.371256515 | 1.15E-13 | 0.432936756 | 0.000550343 |
| HNRNPU | 0.37100022 | 1.20E-13 | 0.510901074 | 3.03E-05 |
| GLA | 0.370915822 | 1.21E-13 | 0.512885238 | 2.79E-05 |
| STT3B | 0.370540414 | 1.29E-13 | 0.43346768 | 0.000540806 |
| BOP1 | 0.369092093 | 1.63E-13 | 0.577467938 | 1.36E-06 |
| PRPF19 | 0.368511519 | 1.79E-13 | 0.428933025 | 0.000627321 |
| DPY30 | 0.368352243 | 1.84E-13 | 0.454090322 | 0.000268153 |
| CABYR | 0.368130784 | 1.90E-13 | 0.469793079 | 0.000152451 |
| EHMT2 | 0.367350853 | 2.16E-13 | 0.544058549 | 7.02E-06 |
| PSMD4 | 0.366610483 | 2.43E-13 | 0.65028516 | 1.87E-08 |
| FAM83D | 0.365755212 | 2.79E-13 | 0.50160469 | 4.45E-05 |
| TTL | 0.3654134 | 2.94E-13 | 0.433032618 | 0.00054861 |
| DAP3 | 0.365236342 | 3.03E-13 | 0.54374946 | 7.12E-06 |
| PHYHIPL | 0.36523619 | 3.03E-13 | 0.471327125 | 0.000144055 |
| PTK2 | 0.365036852 | 3.13E-13 | 0.586501634 | 8.47E-07 |
| EPRS1 | 0.363698904 | 3.87E-13 | 0.53881389 | 8.94E-06 |
| PABPC1 | 0.363660736 | 3.89E-13 | 0.547638347 | 5.94E-06 |
| CSE1L | 0.362567165 | 4.62E-13 | 0.502355607 | 4.32E-05 |
| AATF | 0.362511076 | 4.66E-13 | 0.55336215 | 4.53E-06 |
| CEMIP2 | 0.362167268 | 4.93E-13 | 0.613639041 | 1.86E-07 |
| MAD2L1BP | 0.3608591 | 6.05E-13 | 0.477130599 | 0.000115985 |
| PHF20L1 | 0.359726142 | 7.23E-13 | 0.470790184 | 0.000146944 |
| DHX57 | 0.359090766 | 7.98E-13 | 0.427313754 | 0.000661131 |
| TGS1 | 0.358945579 | 8.16E-13 | 0.50140776 | 4.49E-05 |
| PEX2 | 0.358785693 | 8.37E-13 | 0.534507014 | 1.09E-05 |
| WRNIP1 | 0.35828437 | 9.05E-13 | 0.485930549 | 8.29E-05 |
| MTBP | 0.358210599 | 9.15E-13 | 0.447345685 | 0.00033896 |
| FLVCR1 | 0.357451025 | 1.03E-12 | 0.495971286 | 5.58E-05 |
| AXIN2 | 0.357166104 | 1.08E-12 | 0.493100373 | 6.26E-05 |
| TRMT12 | 0.356260553 | 1.24E-12 | 0.482907428 | 9.31E-05 |
| CCDC138 | 0.354938349 | 1.52E-12 | 0.488902262 | 7.38E-05 |
| COPS5 | 0.353553636 | 1.88E-12 | 0.503240715 | 4.16E-05 |
| C8orf33 | 0.352790996 | 2.11E-12 | 0.52882373 | 1.40E-05 |
| MRPL9 | 0.352310875 | 2.27E-12 | 0.537908802 | 9.32E-06 |
| UBAP2L | 0.352121349 | 2.34E-12 | 0.466958536 | 0.000169154 |
| NARS1 | 0.351639507 | 2.51E-12 | 0.436527216 | 0.000488709 |
| CGREF1 | 0.35150642 | 2.57E-12 | 0.622002231 | 1.13E-07 |
| GNPAT | 0.350944255 | 2.79E-12 | 0.660939517 | 9.07E-09 |
| POLR1A | 0.350885667 | 2.82E-12 | 0.601759398 | 3.67E-07 |
| ZFP69B | 0.35049732 | 2.99E-12 | 0.467593467 | 0.000165273 |
| EID3 | 0.350477951 | 3.00E-12 | 0.450603582 | 0.000302869 |
| HSF1 | 0.349643538 | 3.40E-12 | 0.465626437 | 0.000177568 |
| CHRAC1 | 0.349544293 | 3.45E-12 | 0.53181068 | 1.23E-05 |
| NELFE | 0.349518283 | 3.47E-12 | 0.438368986 | 0.000459588 |
| ARMC1 | 0.349403111 | 3.53E-12 | 0.476048593 | 0.000120804 |
| HEATR1 | 0.349137383 | 3.67E-12 | 0.696195252 | 6.58E-10 |
| ADSL | 0.348240167 | 4.20E-12 | 0.515202556 | 2.53E-05 |
| PARP1 | 0.347731283 | 4.54E-12 | 0.542566987 | 7.53E-06 |
| COPA | 0.347703137 | 4.55E-12 | 0.482898326 | 9.31E-05 |
| MTERF3 | 0.347469643 | 4.72E-12 | 0.473835897 | 0.000131234 |

Supplementary Figure legends

Supplementary Figure 1: Transcriptional level of the 10 hub genes in HepG2 cells with or without BAG2 silencing. All values shown were mean ± SD. P<0.05 was statistically significant, *P<0.05, **P<0.01, ***P<0.001.

Supplementary Figure 1


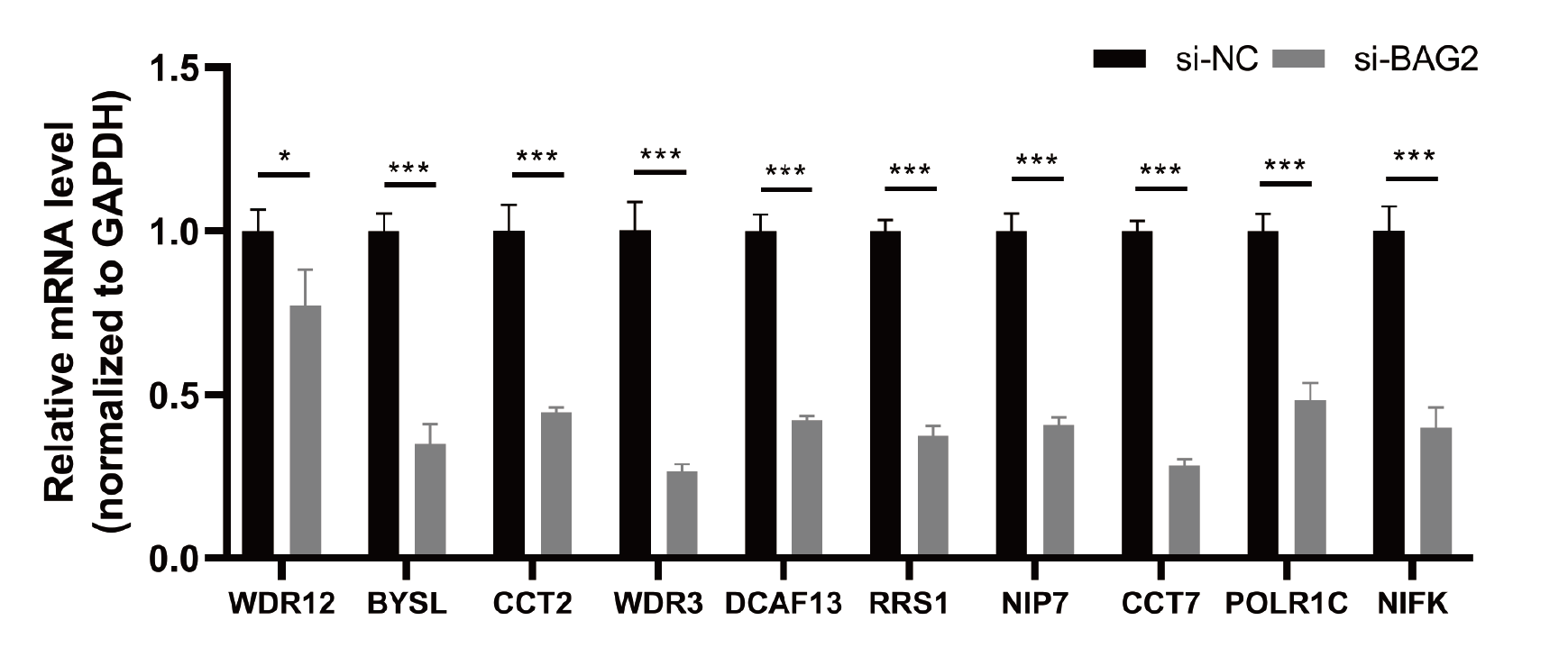

Supplement: Supplementary file 1 [file DataSheet1.docx]
